# Apatinib weakens proliferation, migration, invasion, and angiogenesis of thyroid cancer cells through downregulating pyruvate kinase M2

**DOI:** 10.1038/s41598-023-50369-w

**Published:** 2024-01-09

**Authors:** Xia Yang, Wenhong Li, Xiaoying Han, Jiao Wang, Jianjian Dai, Xin Ye, Min Meng

**Affiliations:** 1 Department of Oncology, Shandong Provincial Hospital Affiliated to Shandong First Medical University, 324 Jingwuweiqi Road, Jinan, 250021 Shandong China; 2 Department of Oncology, The First Affiliated Hospital of Shandong First Medical University, 16766 Jingshi Road, Jinan, 250014 Shandong China

**Keywords:** Cancer, Cell biology, Drug discovery

## Abstract

Thyroid cancer (TC) is the most frequent malignancy of the endocrine system. Apatinib, as an anti-angiogenic agent, has been applied in the therapy of several cancers. However, the function and mechanism of Apatinib in TC have not been clearly elucidated. After processing with Apatinib alone or combined PKM2 overexpression plasmids, cell proliferation, migration, and invasion were analyzed by EdU staining, CCK-8, wound healing, and Transwell. Meanwhile. HUVECs were incubated with the conditioned medium prepared from cell culture medium, and tube formation and VEGFR2 expression in HUVECs were examined using tube formation and immunofluorescence (IF) assays. Besides, we established a nude mouse xenograft model by lentivirus-mediated PKM2 shRNAs, and tested the growth of tumors; the pathological structure was analyzed with H&E staining. And the expressions of N-cadherin, Vimentin, E-cadherin, PKM2, VEGFA, VEGFR2, and Ki67 were determined by immunohistochemistry or Western blot. Apatinib could prominently suppress proliferation, migration, invasion, and HUVEC tube formation in SW579 and TPC-1 cells. Besides, we discovered that Apatinib had a significant inhibitory role on the expression of pyruvate kinase M2 (PKM2) in TC cells. And PKM2 overexpression also could notably reverse Apatinib-mediated inhibition of TC progression. Moreover, PKM2 shRNAs were applied to TC xenografts, resulting in significant reduction in tumor volume and suppression of angiogenesis-related protein expression. In summary, Apatinib has a regulatory role in TC progression, and Apatinib can block cancer cell angiogenesis by downregulating PKM2. This will provide a theoretical basis for therapy of TC.

## Introduction

Thyroid cancer (TC) is a malignant tumor originating from thyroid follicular epithelial cells, accounting for about 1% of all malignant tumors^1^. TC has become the endocrine system malignancy with the fastest increasing incidence worldwide^2^. TC has an insidious onset and its clinical manifestations differ little from those of benign thyroid tumors, so it is easy to be misdiagnosed. For most of the patients with TC, surgery and radioactive iodine therapy can achieve good treatment results^3^. While some patients have only 15–20% 10-year survival rate due to incomplete surgical resection, resulting in postoperative metastasis and recurrence^4^. Besides, some patients are not sensitive to radiotherapy^5^. And radiotherapy kills cancer cells while causing significant damage to healthy cells, resulting in serious toxic side effects^6^. Currently, the invasion and metastasis of tumor cells are the most critical cause of therapeutic failure and death in TC patients^7^. And TC is also insensitive to general chemotherapeutic agents^8^. Therefore, the selection of precise and effective targeted drugs has become the main research direction for TC therapy.

TC microenvironment is mainly composed of tumor cells, immune cells and vascular endothelial cells, whose interactions play a key role in pathological angiogenesis of tumors^9^. Interactions between tumor cells and their neighboring stromal cells are mainly mediated by cytokines or growth factors in a paracrine manner, thereby regulating tumor angiogenesis^10^. Angiogenesis is also the main condition that causes the biological behavior of solid tumors such as malignant proliferation and metastasis^11^. Vascular endothelial growth factor (VEGF), as the most endothelial cell-specific regulatory factor with high ability to build tumor vasculature, has been considered as a molecular marker for tumors^12^. Apatinib is a novel inhibitor of VEGF receptor (VEGFR), which can block the vascular endothelial cell transduction pathway, thereby suppressing proliferation and migration of vascular endothelial cells, lowering tumor microvessel density, and preventing cancer progression^13^. In phase II and III clinical trials, Apatinib has been applied in the therapy of advanced gastric cancer, breast cancer, and lung cancer. It has also been proven that Apatinib has a blocking effect on the TC process^14–16^. However, the mechanism of Apatinib in TC therapy has not been fully elucidated.

Aberrant glucose metabolism is another vital characteristic of tumor cells^17,18^. Normal cells can undergo glycolysis to produce lactate in the absence of oxygen. Whereas tumor cells metabolize energy primarily through glycolysis regardless of the presence or absence of oxygen, which is also called the Warburg effect^19^. Pyruvate kinase M2 (PKM2), a key enzyme in the glycolytic pathway, was connected with increased glucose uptake and lactic acid production, and decreased oxygen consumption in tumor cells^20,21^. Study also verified that PKM2 was relevant to the processes of multiple tumor cells, such as breast cancer^22^, bladder cancer^23^, hepatocellular carcinoma^24^, cervical cancer^25^, etc. However, the role and mechanism of PKM2 in the TC progression has not been reported. In our pre-experiments, we fortunately discovered that Apatinib could notably downregulate PKM2 in TC cells. This suggested that Apatinib may exert its oncogenic effect by downregulating PKM2 in TC cells (Supplementary Fig. 1).

In this study, we investigated the antitumor effects of Apatinib on TC, and its regulation of PKM2 expression, with the aim of providing data to support the elucidation of Apatinib for TC therapy.

## Results

### Apatinib markedly restrained proliferation, migration, and invasion of SW579 and TPC-1 cells

**Figure 1:**
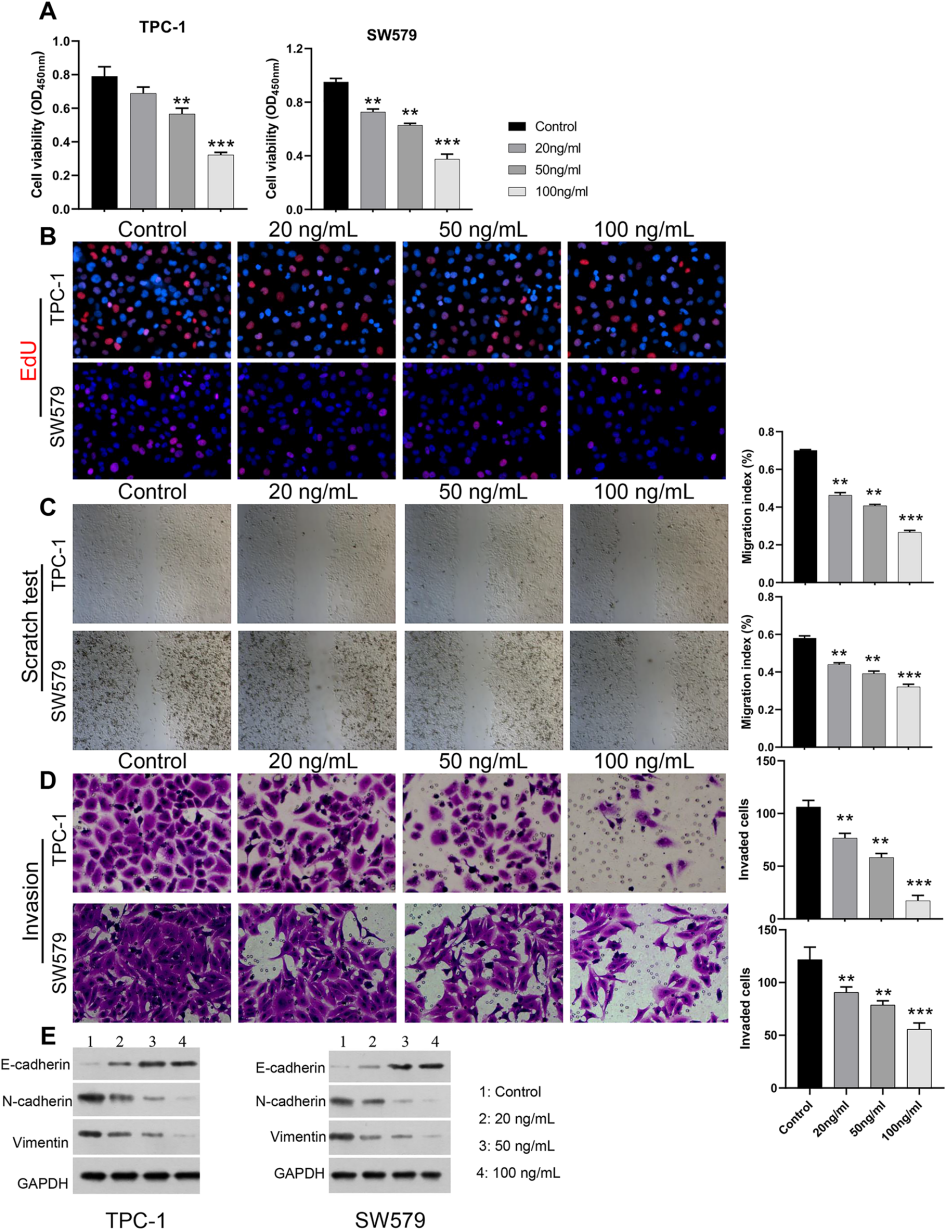
Apatinib markedly restrained proliferation migration, and invasion of SW579 and TPC-1 cells. (**A**) CCK8 was used to measure the cell proliferation of SW579 and TPC-1 cells. *P < 0.05, **P < 0.01, vs. Blank. (**B**) EdU staining was used to detect cell proliferation of of SW579 and TPC-1 cells. (**C**) Wound healing demonstrated the change of cell migration ability in Apatinib-treated SW579 and TPC-1 cells. Magnification, 100 ×. (**D**) Cell invasion was evaluated through Transwell assay in SW579 and TPC-1 cells after processing with Apatinib. (**E**) Western blot was utilized to analyze the change of N-cadherin, Vimentin, E-cadherin expressions in SW579 and TPC-1 cells after administration with Apatinib. Magnification, 200 ×.

We first investigated the impacts of Apatinib on malignant biological properties of TC cells. As demonstrated in Fig. 1A, after treatment with 50 and 100 ng/ml Apatinib, CCK-8 data manifested the proliferation activity of SW579 and TPC-1 cells was observably lower than that of the control group. Similarly, EdU labelling assay showed that introduction of Apatinib significantly reduced the proliferation viability of SW579 and TPC-1 cells, and the cell proliferation capacity gradually decreased with the increase of Apatinib concentration (Fig. 1B). Meanwhile, wound healing results signified that relative to the control group, the migratory ability of SW579 and TPC-1 cells was markedly reduced after Apatinib treatment, especially at high concentration of Apatinib (Fig. 1C). And Transwell data denoted that cell invasion was significantly lower in the Apatinib-treated group than in the control group, and the inhibition effect was strongest in the high concentration group (Fig. 1D). Western blot results for EMT-related markers showed that Apatinib can upregulate the protein expression of epithelial phenotypic markers (E-cadherin), while downregulating the mesenchymal phenotypic markers (N-cadherin and Vimentin) (Fig. 1E). These data revealed that Apatinib has a significant blocking effect on the malignant process of TC cells.

### Apatinib prominently suppressed HUVEC tube formation and weakened PKM2 expression in TC

**Figure 2:**
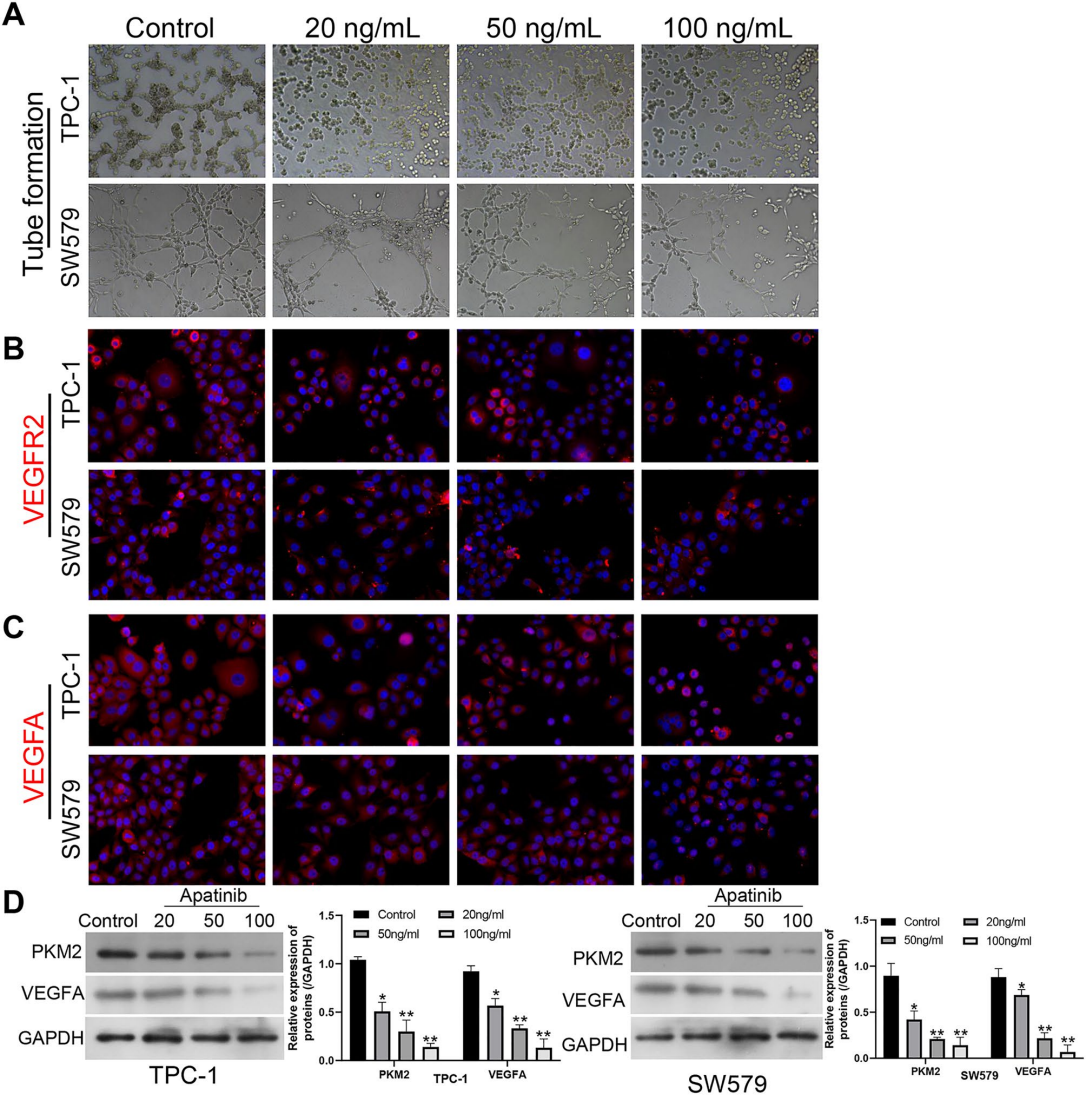
Apatinib prominently suppressed HUVEC tube formation and weakened PKM2 expression in TC. (**A**) Images of tube formation assay in HUVECs, which were incubated with the conditioned medium from Apatinib-treated SW579 and TPC-1 cells. Magnification, 100 ×. (**B**) After incubation with the conditioned medium, IF staining was conducted to assess the expression change of VEGFR2 in HUVECs. Magnification, 200 ×. (**C**) The expression change of VEGFA was credited through IF staining in HUVECs after treatment with the conditioned medium from Apatinib-processed SW579 and TPC-1 cells. Magnification, 200 ×. (**D**) Western blot was utilized to analyze the change of VEGFA and PKM2 expressions in SW579 and TPC-1 cells after administration with Apatinib.

Furthermore, we examined the influence of Apatinib on HUVEC tube formation. We collected the supernatant of SW579 and TPC-1 cells after Apatinib treatment and made the supernatant into tumor-conditioned medium to incubate HUVECs. And the data uncovered that tube formation ability of HUVECs was dramatically decreased after processing with the conditioned medium from Apatinib-treated SW579 and TPC-1 cells relative to Apatinib-untreated cells (Fig. 2A). And IF staining results disclosed that relative to control group, the expression of VEGFR2 was also notably lowered in HUVECs after treatment with the conditioned medium from Apatinib-treated SW579 and TPC-1 cells (Fig. 2B). Next, we further tested the expression change of VEGFA in HUVECs using IF staining. As displayed in Fig. 2C, VEGFA expression was signally decreased in Apatinib group compared to that in the control group. Besides, we discovered that Apatinib memorably downregulated PKM2 and VEGFA in SW579 and TPC-1 cells through western blot (Fig. 2D). In this way, we demonstrated that Apatinib has a prominent inhibitory role on angiogenesis, and Apatinib also could downregulate PKM2 in TC cells. This also suggests that PKM2 has a vital regulatory role in Apatinib-mediated TC progression. And on account of the effect of different concentrations of Apatinib, we chose 50 ng/ml for the follow-up study.

### PKM2 overexpression notably reversed apatinib-mediated suppression of proliferation, migration, and invasion in SW579 and TPC-1 cells

**Figure 3:**
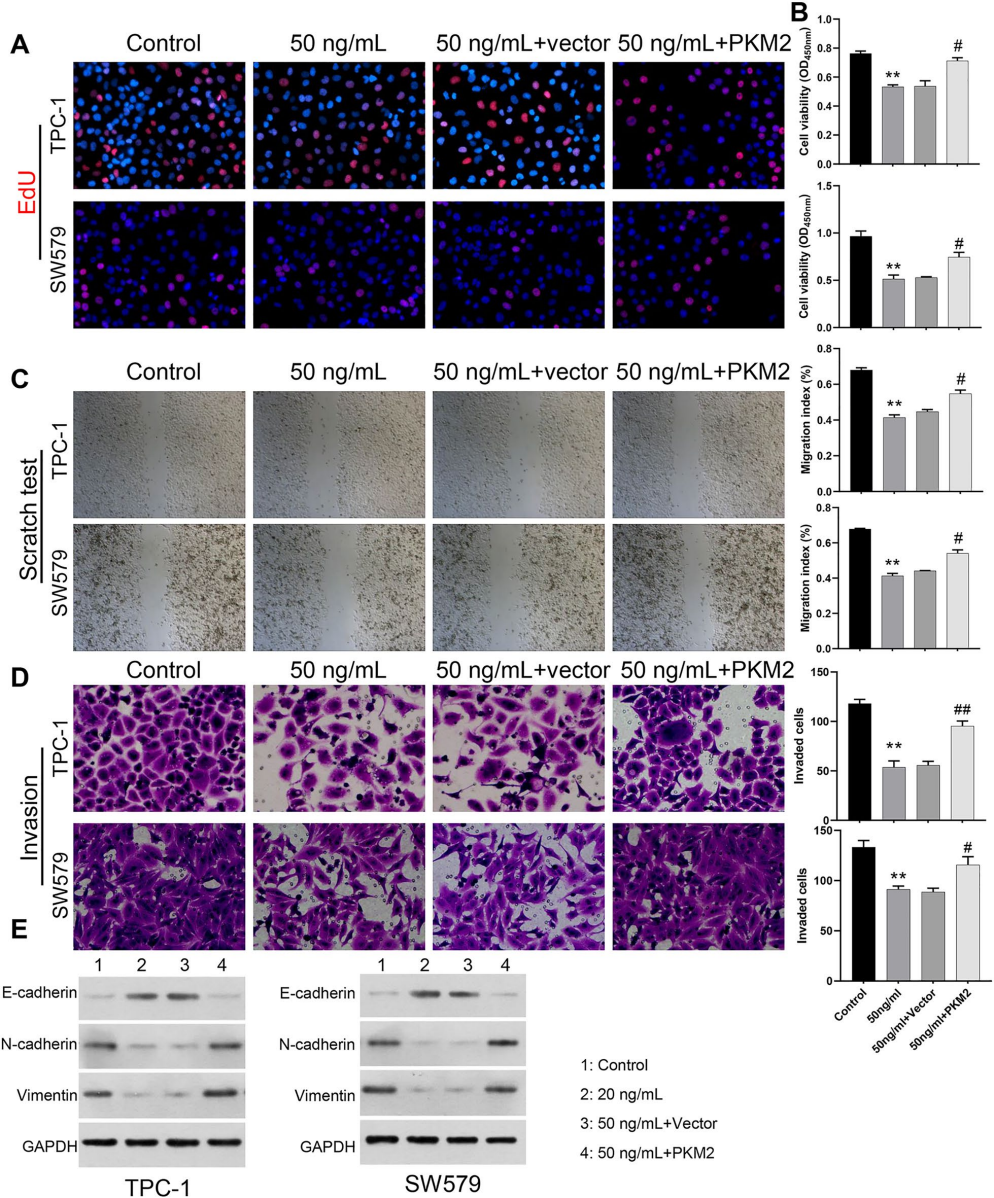
PKM2 overexpression notably reversed Apatinib-mediated suppression of proliferation, migration, and invasion in SW579 and TPC-1 cells. (**A**) SW579 and TPC-1 cells were treated with Apatinib or/and PKM2 overexpression plasmid, EdU staining was conducted to examine the change in cell proliferative activity. Magnification, 200 ×. (**B**) CCK8 was used to measure the cell proliferation of SW579 and TPC-1 cells. **P<0.01 VS. Control. #*P<0.05 vs. 50ng/mL + vector.* (**C**) Cell migration was credited via wound healing. Magnification, 100 ×. (**D**) Transwell presented the change in cell invasion capacity in each group. (**E**) Western blot displayed the change levels of N-cadherin, Vimentin, E-cadherin expressions in SW579 and TPC-1 cells. Magnification, 200 ×.

In line with the above study, we further verified the roles of PKM2 in the malignant progression of TC mediated by Apatinib. We overexpressed PKM2 in Apatinib-treated SW579 and TPC-1 cells. EdU staining data exhibited that the inhibitory effect of Apatinib on the proliferative activity of SW579 and TPC-1 cells could be prominently reversed by PKM2 overexpression (Fig. 3A). Subsequently, the CCK8 assay further verified that the overexpression of PKM2 significantly enhanced the Apatinib-reduced cell proliferation observed in SW579 and TPC-1 cells (Fig. 3B). Wound healing results indicated that PKM2 overexpression observably attenuated the blocking role of Apatinib on the migration of SW579 and TPC-1 cells (Fig. 3C). And Transwell data also represented that PKM2 overexpression also could markedly enhance the invasion capacity of SW579 and TPC-1 cells, which was suppressed by Apatinib (Fig. 3D). Furthermore, overexpression of PKM2 triggered EMT that is inhibited by Apatinib, via enhancement of N-cadherin, Vimentin and inhibition of E-cadherin in SW579 and TPC-1 cells (Fig. 3E). Thus, we demonstrated that Apatinib can prevent TC process by downregulating PKM2.

### PKM2 overexpression dramatically attenuated the inhibitory effect of Apatinib on HUVEC tube formation in SW579 and TPC-1 cells

**Figure 4:**
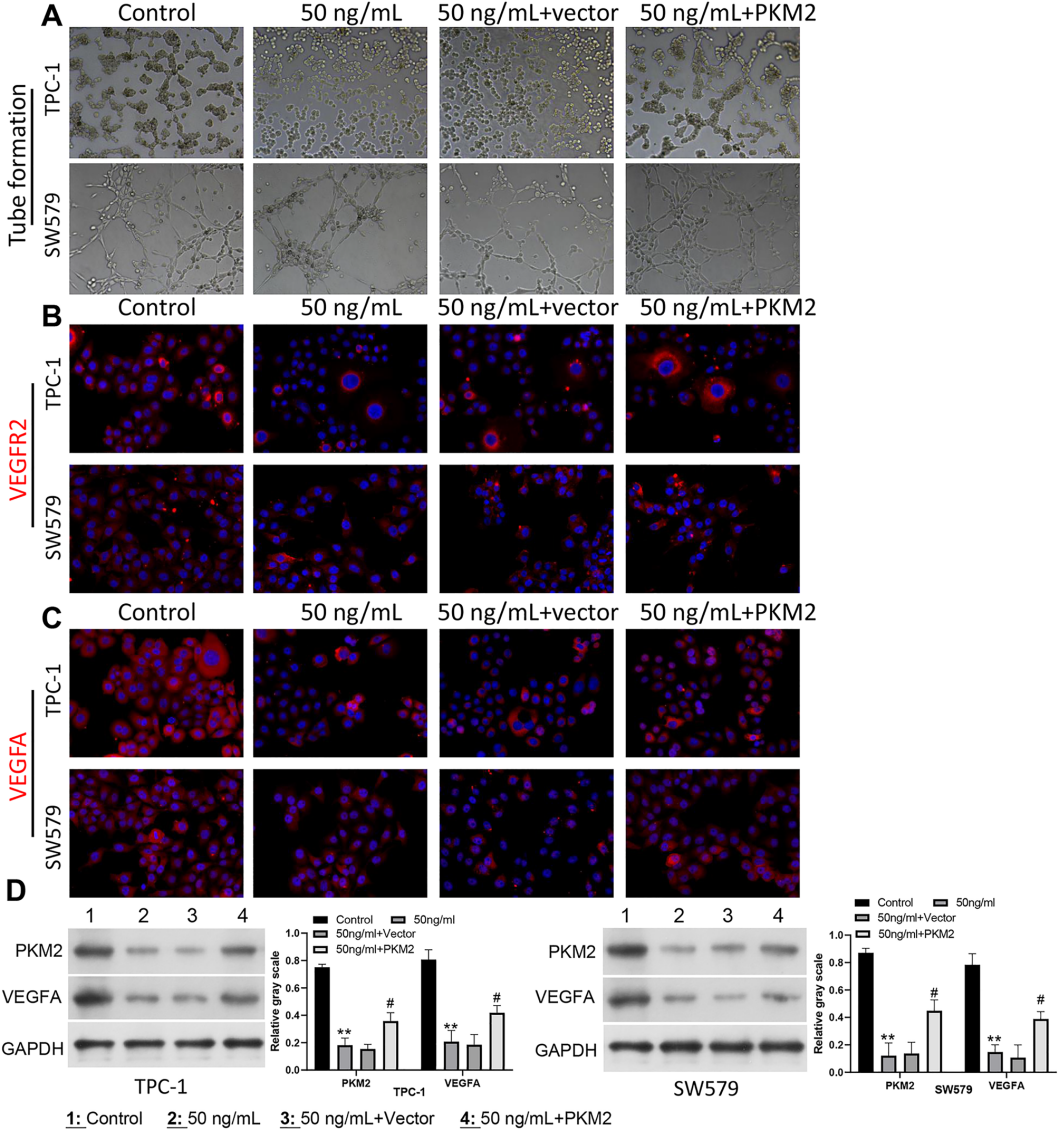
PKM2 overexpression dramatically attenuated the inhibitory effect of Apatinib on HUVEC tube formation in SW579 and TPC-1 cells. (**A**) Apatinib-processed SW579 and TPC-1 cells were then transfected with PKM2 overexpression plasmid. After incubation with the conditioned medium from the treated SW579 and TPC-1 cells, the angiogenesis was assessed by applying tube formation assay in HUVECs. Magnification, 100 ×. (**B**) The change in VEGFR2 expression was examined through IF staining in the processed HUVECs. Magnification, 200 ×. (**C**) IF staining was adopted to evaluate the change of VEGFA expression in each group. Magnification, 200 ×. (**D**) Western blot displayed the change levels of PKM2 and VEGFA expressions in SW579 and TPC-1 cells.

Additionally, we also studied the influence of PKM2 overexpression on HUVEC tube formation. HUVECs were incubated with the tumor-conditioned medium of SW579 and TPC-1 cells, which have been processed with Apatinib and PKM2 overexpression plasmid. The results from tube formation assay signified that Apatinib memorably reduced the angiogenesis capacity of HUVECs, which also could be notably restored by PKM2 overexpression in SW579 and TPC-1 cells (Fig. 4A). Meanwhile, the data of IF staining represented that addition of Apatinib in SW579 and TPC-1 cells signally reduced the expression of VEGFR2 in HUVECs, which also could be dramatically weakened by PKM2 overexpression (Fig. 4B). Similarly, IF result also indicated that Apatinib could prominently downregulate VEGFA by PKM2(Fig. 4C). In addition, western blot data manifested that PKM2 overexpression could prominently attenuate the downregulation of PKM2 and VEGFA expressions mediated by Apatinib (Fig. 4D). Overall, this part of the data confirmed that Apatinib can prevent angiogenesis of HUVECs by downregulating PKM2.

### PKM2 silencing prevented subcutaneous tumor growth and angiogenesis in a nude mouse TC model

**Figure 5:**
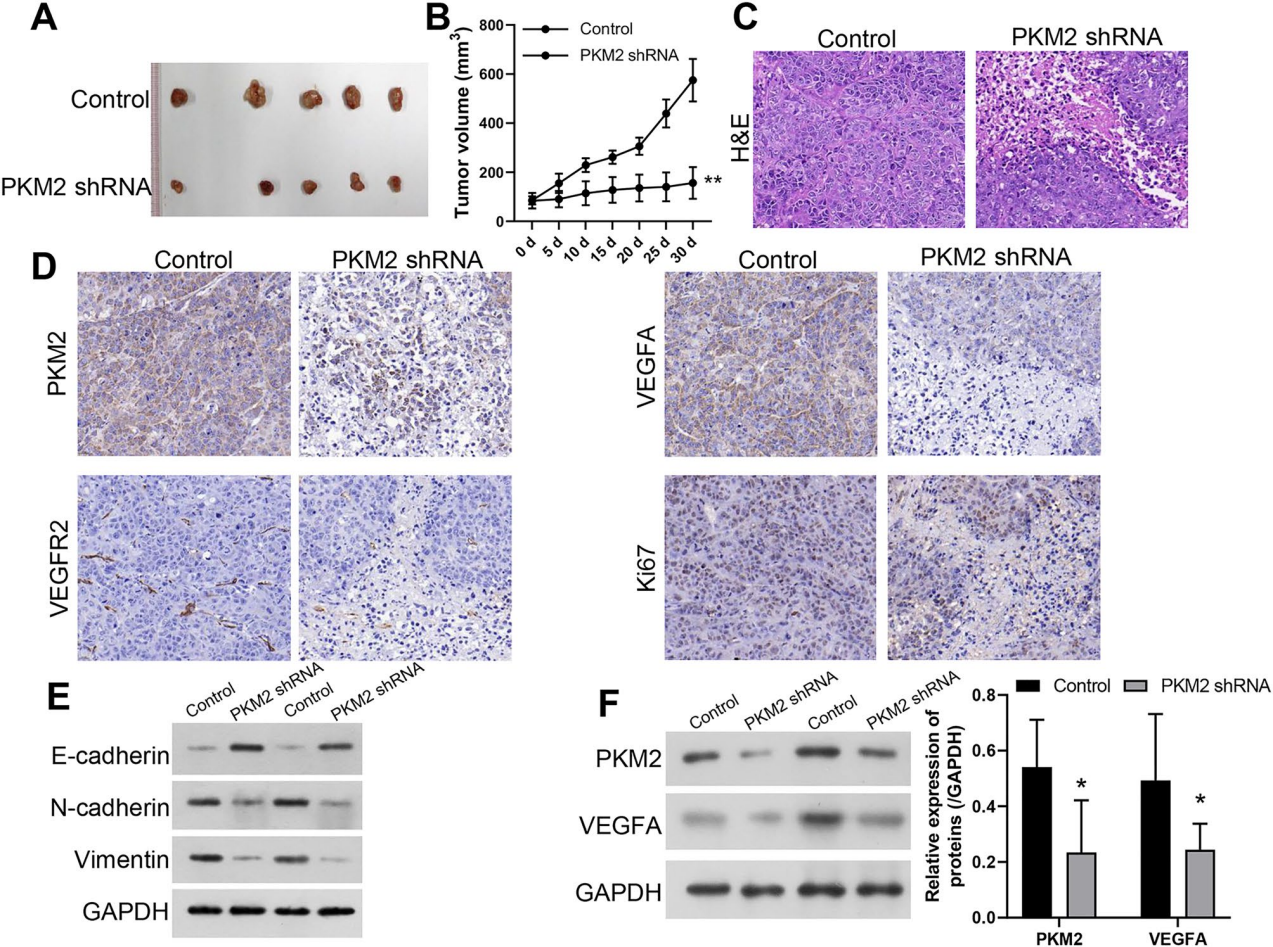
PKM2 silencing prevented subcutaneous tumor growth and angiogenesis in a nude mouse TC model SW579 cells stably transfected with PKM2 shRNAs were subcutaneously injected into nude mice. (**A**) Subcutaneous tumor cells were excised and displayed. (**B**) Tumor volume was tested and calculated every 5 days for 30 days. (**C**) H&E staining presented the change in pathological structure. Magnification, 200 ×. (**D**) IHC assay was applied to certify the changes in PKM2, VEGFA, VEGFR2, and Ki67 expressions in each group of tumors. Magnification, 200 ×. (**E**) The expression changes of N-cadherin, Vimentin, E-cadherin were tested using western blot in tumors. (**F**) The expression changes of PKM2 and VEGFA were tested using western blot in tumors.

As proved by the in vitro results, PKM2 overexpression could induce proliferation, metastasis and HUVEC tube formation of Apatinib-treated SW579 and TPC-1 cells. We further confirmed the impacts of PKM2 silencing on the growth, pathologic structure, and angiogenesis of TC grafted tumor in nude mice. As denoted in Fig. 5A,B, relative to the control group, the tumor was markedly reduced in the PKM2 silencing group. Then H&E staining results displayed that in the control group, thyroid tumor cells of nude mice had normal and clear outlines, abundant cytoplasm, obvious nucleoli and heterotypy; in the PKM2-silenced group, thyroid tumor cells were seen to have necrotic cells, increased nuclei division and blurred cell outlines; the number of necrotic thyroid cells in the PKM2-silenced group was reduced compared with that in the control group (Fig. 5C). Besides, IHC data indicated that knockdown of PKM2 could lower PKM2, VEGFA, VEGFR2, and Ki67 expressions in the grafted tumors (Fig. 5D). Simultaneously, western blot results also showed that PKM2 knockdown could lead to a remarkable downregulation of N-cadherin, Vimentin, PKM2, and VEGFA expression in tumor tissues, while E-cadherin expression was notably upregulated (Fig. 5E,F). So, these data testified that PKM2 knockdown had a significant attenuating effect on the development of TC in vivo.

## Discussion

TC has a high incidence and its causative mechanisms have not been specifically elucidated clinically^4^. On account of the literatures, the causes of TC are relevant to radiation, genetics, poor lifestyle habits, and abnormal iodine intake^26^. Currently, most TC have slow progression and can achieve good prognosis and long survival time after standard treatment^27^. However, some patients still have the risk of tumor recurrence, metastasis or death. Meanwhile, advanced TC is more difficult to treat and has a poor prognosis^28^. Therefore, it is extremely key to take timely and effective measures for the therapy of patients with advanced TC.

A key feature of tumor growth and metastasis is aberrant angiogenesis, in which the vascular endothelial growth factor (VEGF) pathway plays a key role^29^. Research proved that tumor vascular endothelial cells can secrete VEGF, which can participate in the generation of new microvessels and cause an imbalance of endothelial growth and inhibition^30^. The formation of nascent microvessels can also create a hypoxic environment, which induces VEGF transcription and exacerbates tumor microangiogenesis^31^. VEGF receptor (VEGFR) is the key receptor in VEGF pathway^32^. Overexpression of VEGF-A, VEGFR1 and VEGFR2 has been observed in more than 90% of TC patients^33,34^. VEGFR activation can accelerate endothelial cell proliferation, survival, migration and invasion, thereby increasing vascular permeability to induce tumor angiogenesis^35^. Research proved that high expression of VEGF and VEGFR was associated with tumor growth, metastasis, microvessel density, and poor patient prognosis in TC^36^. Suppression of tumor angiogenesis has become a novel strategy for targeted tumor therapy. Currently, anti-angiogenic drugs have made significant advances in TC therapy. However, there are multiple disadvantages of anti-angiogenic drug, such as expensive, unstable efficacy, large adverse effects, and unknown mechanisms of drug resistance, etc. Therefore, anti-angiogenic targeted therapies in TC still need to be studied in depth.

Apatinib, as anti-angiogenic targeted drug, is vital for the clinical therapy of malignant tumors^37^. Research demonstrated that Apatinib can selectively compete with VEGFR2 binding sites in cells to prevent angiogenesis in tumor tissues^38^. Multiple researches also demonstrated the potential function of Apatinib in cancer progression^39,40^. In recent years, Apatinib has also been reported in TC. For instance, researches suggested that Apatinib can induce autophagy and apoptosis in human TC cells^16^; Apatinib has an inhibitory effect on angiogenesis of TC cells^41^. In our study, we further testified that Apatinib could obviously suppress proliferation, migration, invasion, as well as HUVEC tube formation of TC cells. and Apatinib also could downregulate VEGFR2 and VEGFA in TC. Thus, we further confirmed the noteworthy blocking effect of Apatinib on the malignant process of TC.

Tumor cells adopted aerobic glycolysis as the main mode of energy supply^18,42^. Pyruvate kinase (PK), a key enzyme in the glycolysis, can catalyze the production of pyruvate from phosphoenolpyruvate^43^. Among them, PKM2 is one of the isozymes of PK and displays overexpression in multiple of tumor cells^21,44^, and exists primarily as an enzymatically inactive monomer or dimer. Some evidences indicated that nuclear PKM2 could promote the Warburg effect and cell cycle progression in cancer cells and contributes to tumorigenesis^45^. Research certified that tumor cells can acquire the metabolic property of preferential glycolysis by expressing PKM2, which also enables them to obtain vast energy to maintain their high proliferation and metastasis^21^. Besides, PKM2 can also affect the growth of tumor cells by regulating the products and of cellular metabolic processes and related cytokines^46^. However, there are no reports about PKM2 in TC. In our study, we data also Apatinib memorably downregulated PKM2 in TC cells. Moreover, we proved that PKM2 is required for Apatinib-mediated inhibition of TC cell proliferation, metastasis, and angiogenesis. And we disclosed that PKM2 knockdown could prevent subcutaneous tumor growth and angiogenesis in vivo. The PKM2 dimer is capable of migrating into the nucleus, where interacts directly with the HIF-1α and regulate expression of numerous pro-glycolytic enzymes^47^. Lactate, as an end product of glycolysis, stimulates angiogenesis by increasing the production of VEGF and VEGFR2 in HUVECs^48^. Thus, this could elucidate how Apatinib leads to decreased expression of VEGF by inhibiting PKM2 and consequently inhibits angiogenesis.

Lin et al.^49^ have reported in their phase II clinical trial that Apatinib has shown therapeutic efficacy in radioiodine-refractory differentiated thyroid cancer (RAIR-DTC), achieving an objective response rate (ORR) of 80% and a disease control rate (DCR) of 95%. The study by Du et al.^50^ has also found that the patients undergoing Apatinib for RAIR-DTC, have achieved an ORR of 80% and a DCR of 90%. Zhang et al.^14^ have reported a case of an inoperable locally advanced DTC patient, who has undergone a curative operation after the treatment of preoperative monotherapy of apatinib. Currently, most TC-related studies have focused on the clinical efficacy observation and safety analysis of Apatinib and its combination with other drug therapy, while the mechanism of anti-cancer effect of Apatinib has been less studied. Our study is the first to demonstrate that Apatinib can achieve anti-TC effects by altering PKM2 expression in tumor cells. This might provide a new idea for the antitumor role of Apatinib.

## Materials and methods

### Cell culture

SW579 and TPC-1 cells were purchased from the ATCC. HUVECs were purchased from ScienCell (USA). SW579 cells were incubated in L-15 medium (GIBCO, Cat. no. 41300039), and TPC-1 cells grown in Minimum essential medium (MEM; GIBCO, Cat. no. 41500034). HUVECs were cultured in ECM medium (Life Technologies). The cultures all contained 10% fetal bovine serum (Gibco, USA). And both types of cells were all incubated in an incubator at 37 °C with 5% CO_2_.

### Cell treatment

SW579 and TPC-1 cells were first disposed of 20 ng/ml, 50 ng/ml, 100 ng/ml Apatinib (Selleck Chemicals; cat. no. S5248) for 48 h^51^. PKM2 overexpression plasmid and empty vector, PKM2 shRNAs and control were provided by Integrated Biotech Solutions (Shanghai, China). SW579 and TPC-1 cells were (density about 60%) in 6-well plate were transfected with PKM2 overexpression plasmid and vector by applying Lipofectamine 3000 (Invitrogen) in accordance with the reagent instructions. PKM2 shRNAs lentivirus was packaged by WZ Bioscience (Shandong, China). And lentivirus was applied to infect SW579 cells at MOI = 50, and then added with 5 mg/ml Polybrene (Santa Cruz). After 96 h of infection, cells were added with 4 μg/ml Puromycin to screen stable expression cells.

### EdU staining

BeyoClickTM EdU Proliferation Kit (Beyotime, Shanghai, China) was applied for this experiment. After increasing with 10 μL EdU solution, the treated SW579 and TPC-1 cells were incubated at 37 °C for 3 h. After washing, cells were fixed by adding 4% paraformaldehyde for 15 min and incubated with 0.5% TritonX-100 for 20 min. After rinsing, SW579 and TPC-1 cells were dyed with Hoechst33342 (Sigma-Aldrich) for 30 min protected from light. After washing, the results were photographed under a fluorescence microscope, and EdU-positive cells were counted.

### CCK-8

SW579 and TPC-1 cells were prepared into a 2 × 10^5^/ml cell suspension, which were then uniformly inoculated into a 96-well plate (100 µl per well). Then cells were processed depending on the purpose of the study. 10 µl CCK-8 reagent (Beyotime) was applied to treat cells in each well at 48 h. After 3 h, a microplate reader was utilized to test the optical density (OD) values of each well at 450 nm.

### Wound healing

SW579 and TPC-1 cells (1 × 10^6^ cells/ml) were evenly inoculated into 6-well plate, and processed in line with the experiment purpose. When the cell coverage of the monolayer reached about 90%, a straight line was scratched with 10 μl of gun tip. cells were washed with PBS and continued to be cultured with serum-free medium for 24 h. The scratch wound assay was photographed at 0 h and 24 h, respectively. The migration index was quantified using the following equation: (scratch distance at 0 h − scratch distance at 24 h)/scratch distance at 0 h. Each experiment was performed in triplicate.

### Transwell

The serum-free medium was diluted with Matrigel (EMD Millipore; Cat. no. 356234) at 8:1, and 200 μl of the mixture was placed on the upper level of Transwell chambers (8 μm, Corning) at 37 °C for 60 min. Each group of cells was collected after processing and their concentration was adjusted to 1 × 10^5^ cells/ml. The 200 μl cell suspension was inoculated in the upper chamber of Transwell, and the lower chamber was supplemented with complete medium. After 24 h incubation, the upper matrix and cells were removed, and the lower cells were fixed through 4% paraformaldehyde (15 min) and stained using 1% crystal violet (10 min). After washing using distilled water, the invaded cells were observed under a light microscope. random fields were selected and photographed for counting (200 ×).

### Preparation of conditioned medium

SW579 and TPC-1 cells were addressed for 48 h based on the experimental purpose, and the medium was collected. Then the medium was centrifuged to obtain the tumor cell culture supernatant. Tumor conditioned medium was made in the ratio of tumor supernatant: medium: FBS of 4:5:1.

### Tube formation assay

Referring to the related study^52^, HUVECs (3 × 10^4^ cells) first were inoculated in 6-well plates and incubated for 72 h (5% CO_2_, 37 °C) to achieve 50% coverage. Subsequently, HUVECs were added with the conditioned medium and incubated continuously for 48 h (5% CO_2_, 37 °C). Then 50 μl of Matrigel was added to 96-well plates and polymerized for 30 min at 37 °C. Groups of HUVECs (2 × 10^4^/well) were inoculated onto Matrigel-coated 96-well plates. After incubation for 10 h at 37 °C, HUVECs were observed under a microscope (OLYMPUS CH-2) and the formation of capillary-like structures was assessed.

### Immunofluorescence staining

The treated SW579 and TPC-1 cells were inoculated on cover glass containing polylysine and cultured at 37 °C for 24 h. Then the cells were fixed using 4% paraformaldehyde for 20 min and permeated using 0.2% Triton-X-100 for 10 min. After being sealed with 1% bovine serum albumin (BSA) for 2 h, the cells were exposed to primary antibody (Abcam) at 4 °C overnight, followed by fluorescent labeled IgG (secondary antibody) for 2 h. Subsequently, the cells were addressed with DAPI for 15 min. Finally, localization and expression of VEGFR2 and VEGFA proteins in cells were observed under fluorescence microscope (the cells were washed with pre-cooled PBS for 3 times, 5 min each time).

### Western blot

The processed cells or tumor tissue after grinding at low temperature were fully lysed by adding RIPA (Sigma-Aldrich, Merck KGaA) and centrifuged with 15,000×*g*/min at 4 °C for 15 min, and the supernatant was retained to obtain total protein. The BCA method (Beyotime) was adopted to determine protein concentration. Protein was added to the loading buffer and cooked at 99 °C for 5 min to denature. Then 40 μg of total protein was separated by 10% SDS-PAGE, and electrotransferred to PVDF membrane (Beyotime, Inc, China). After appropriate tailoring, the membranes were closed with 5% BSA for 1 h and incubated with primary antibodies (anti-VEGFA, anti-PKM2, and anti-GAPDH) overnight at 4 °C. The following day, the membranes were exposed to HRP-labeled secondary antibodies (Abcam) for 1 h after washing. The protein was visualized using Pierce™ ECL substrate (Thermo Fisher Scientific). Image J software (National Institutes of Health, Bethesda, MD, USA) was used to calculate the gray values, and the values were normalized to the GAPDH level to obtain the relative gray value of each protein.

### Subcutaneous tumorigenesis experiment in nude mice

SPF grade BALB/C nude mice (male 5–8 weeks, weighing 15–20 g) were cultured in SPF grade animal center.

The Institutional Animal Care and Use Committee (Shandong Provincial Hospital Affiliated to Shandong First Medical University) approved all studies. Animal experiments conformed to the NIH guidelines (Guide for the Care and Use of Laboratory Animals). All authors complied with the ARRIVE (Animal Research: Reporting of In Vivo Experiments) guidelines. All mice were grown under circadian light (12 h/12 h) at (22 ± 2) °C with freely available clean water and feed. PKM2-silenced SW579 cells (100 μl, 2 × 10^7^ cells/ml) were then injected subcutaneously into the right axilla of mice to construct a mouse TC model. Length (a) and width (b) of the tumor were examined every 5 days for 30 days, and the tumor volume was calculated with V = a × b^2^/2. At the end of the experiment, the mice were euthanized with inhalation of CO_2_, and the tumor was removed and weighed.

### Immunohistochemistry (IHC)

Nude mice in each group were executed by neck amputation. The tumors were removed, fixed in 10% neutral formalin, and then subjected to paraffin embedding, conventional dehydration, transparency, embedding, and serial sectioning (4 μm thick). Subsequently, the sections were dewaxed in xylene, placed in gradient ethanol for dehydration, soaked in 3% H_2_O_2_, and heated in 0.01 mmol/l citrate buffer for antigen repair (130 °C). And the sections were placed in a wet box and supplemented with 10% normal goat serum at 37 °C for 20 min, followed by the diluted antibody (dilution 1:200, Abcam) overnight at 4 °C. Next day, the sections were titrated with secondary antibodies (Abcam) at 37 °C for 30 min. And the sections were again subjected to a series of treatments including DAB color development, hematoxylin eosin re-staining for 2 min, conventional dehydration with hydrochloric acid alcohol, transparency, and sealing. The data were observed and photographed under a light microscope.

### Haematoxylin and eosin (H&E) staining

As treated in IHC, the tumor tissue of each group was embedded, sliced, dewaxed and rehydrated. Then sections were dyed with Hematoxylin and immersed in Eosin. After washing, the sections were dehydrated in gradient alcohol and transparent with xylene. After fixing with neutral gum, the sections were photographed using a light microscope for pathological observation. the sections were assessed by 2 experienced pathologists through double-blind reading.

### Statistical analysis

All experimental data were analyzed using SPSS 23.0 software (Inc., Chicago, IL), and the data were represented as mean ± SD. One-way analysis of variance (ANOVA) was adopted for the comparisons of multiple groups, and Student’s *t* test was adopted for the comparisons between two groups. The test level was 0.05, and *P* < 0.05 signified the statistical significance. All experiments were repeated for three times at least.

### Supplementary Information

Supplementary Figure 1.

## Data Availability

All data generated or analyzed during this study are included in this published article.
