# Supplementary material for: Apatinib weakens proliferation, migration, invasion, and angiogenesis of thyroid cancer cells through downregulating pyruvate kinase M2
Source: Sci Rep. 2024 Jan 9;14:879. doi: 10.1038/s41598-023-50369-w (PMC10776835; doi:10.1038/s41598-023-50369-w)
Supplement: Supplementary file 1 — Supplementary Figure 1. [file 41598_2023_50369_MOESM1_ESM.pdf]

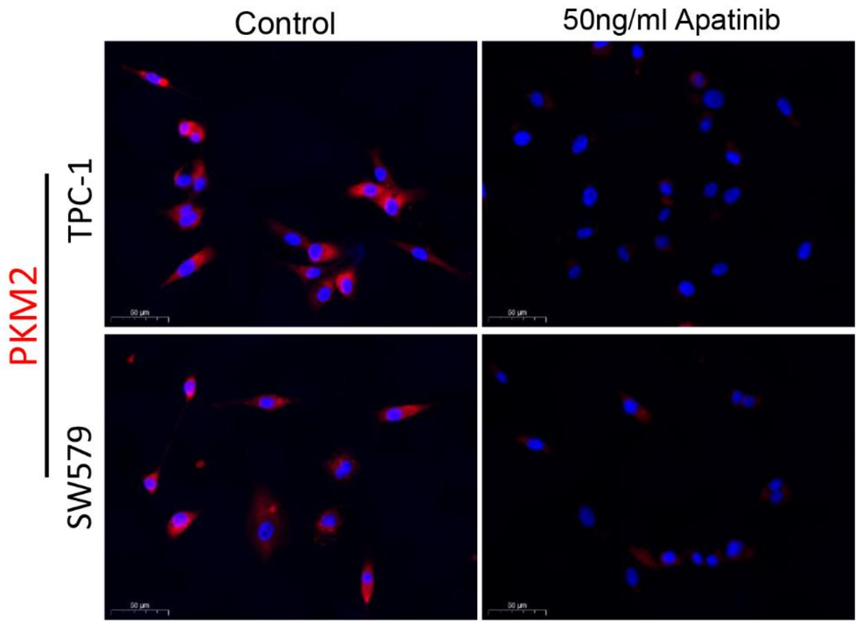

Supplementary Fig 1. The change in PKM2 expression was examined through IF staining in SW579 and TPC-1 cells after treatment with 50 ng/ml Apatinib. Magnification, 200×.
